# Effects of temporary access to environmental enrichment on measures of laboratory mouse welfare

**DOI:** 10.1038/s41598-024-65480-9

**Published:** 2024-07-02

**Authors:** A. S. Ratuski, L. Améndola, I. J. Makowska, D. M. Weary

**Affiliations:** UBC Animal Welfare Program, Faculty of Land and Food Systems, University of British Columbia, Vancouver, Canada

**Keywords:** Laboratory animals, Animal welfare, Environmental enrichment, Abnormal behaviour, Housing, Refinement, Animal behaviour, Behavioural methods, Model vertebrates

## Abstract

Laboratory mice are typically housed in “shoebox” cages with limited opportunities to engage in natural behaviour. Temporary access to environments with increased space and complexity (playpens) may improve mouse welfare. Previous work by our group has shown that mice are motivated to access and use these environments, but it is unknown how other aspects of welfare are impacted. Female C57BL/6J, BALB/cJ, and DBA/2J mice (n = 21; 7 mice per strain) were housed in mixed-strain trios and given temporary access to a large playpen with their cage mates three times per week. Control mice (n = 21; 7 mice per strain) remained in their home cages. Home cage behaviour (development of stereotypic behaviour over time, aggression following cage-changing) and anxiety tests were used to assess how playpen access impacted welfare. Contrary to our predictions, we found increased time spent performing stereotypies in playpen mice; this difference may be related to negative emotional states, increased motivation to escape the home cage, or active coping strategies. Playpen access resulted in strain-dependent improvements in aggression and some measures of anxiety. Aggression was lower for C57BL/6J mice in the playpen treatment following cage changing than it was for C57BL/6J control mice, while playpen mice, and particularly the C57BL/6J strain, spent more time in the center of the open field test and produced fewer fecal boli during anxiety testing, supporting other research showing that strain differences play an important role in behaviour and stress resiliency.

## Introduction

Laboratory mice are typically housed in “shoebox” cages that lack behavioural opportunities, but persist because of convenience, tradition, and the financial investment that would be required to shift to larger housing. Mice housed in shoebox cages show signs of compromised welfare that can be improved to some degree by access to “enrichment” (i.e., additional resources or more complex environments). For example, conventionally housed mice show more agonistic behaviour than those housed in more complex environments^1,2^. Inactive-but-awake behaviour (when a mouse is awake but motionless), associated with depressive states, is also higher in mice living in conventional cages^1,3^. Mice housed in conventional cages show more signs of anxiety^4,5^ and engage in more stereotypic (i.e. abnormal, repetitive) behaviour than mice with access to more environmental complexity^6–8^.

Stereotypic behaviour, aggression, and anxiety are commonly regarded as problematic or undesirable for animal welfare. Reduced stereotypic behaviour is a commonly cited goal of environmental enrichment for rodents^9^. Although the relationship between stereotypic behaviour and animal welfare is complex, this behaviour is more likely in deficient captive housing environments^10^. Aggression is one of the most serious welfare concerns for male laboratory mice^11–13^. Aggression is less well studied in female mice, but this is part of their behavioural repertoire and can be modulated by environmental conditions. Female mice from conventional laboratory cages show more aggression than mice provided with additional environmental resources^1,2^. Escalated aggression is an acute welfare concern^12^ and repeated aggressive encounters can cause chronic stress for subordinate mice^14^, perhaps especially in shoebox cages where mice are unable to physically escape and thus de-escalate interactions. Laboratory mice housed in shoebox cages may behave aggressively as a result of heightened responses to stressful stimuli such as cage changing^15^ or tail handling^16^, although these effects are more commonly studied in males. Deficient housing conditions have also been linked to increased fear or anxiety behaviour, with additional effects on stress hormones and immune responses^17^; these effects are concerning for animal welfare and for their potential impact on experimental outcomes.

The scope for meaningful environmental improvements is limited in shoebox cages as these have little space for additional resources^9^. One potential solution is to provide animals with regular but temporary access to larger, more complex environments (i.e. “playpens”;^18^). Playpens have been used for laboratory rats^19^, ferrets^20^, guinea pigs^21^, and non-human primates^22^, with evidence of various welfare benefits including positive affective states, reduced inactivity, and reduced stereotypic behaviour. Preliminary work by our group has shown that conventionally housed female laboratory mice are motivated to access playpens, and that they display a range of active behaviours in these enclosures^23^. However, it is unclear how the welfare of mice is impacted when mice are in their home cages between playpen visits.

Here, we investigated whether giving female laboratory mice regular access to playpens affected their behaviour. We assessed levels of stereotypic and aggressive behaviour in the home cage, and anxiety in the elevated plus maze and open field tests. We predicted that mice given regular access to playpens would show reduced frequencies of aggression and stereotypies, and lower anxiety compared to control mice with no playpen access.

## Methods

This experiment was approved by the University of British Columbia Animal Care Committee (protocol A18-0104), and was performed in accordance with the guidelines of the Canadian Council on Animal Care and the ARRIVE guidelines.

### Animals and housing

Here we report additional data from the same group of female mice used in a previous publication assessing mouse playpen use and behaviour in the first 5 weeks of access^23^. The current paper reports on additional behaviours from these same mice recorded over a longer time period. C57BL/6J (n = 14), DBA/2J (n = 14), and BALB/cJ (n = 14) mice were obtained from Jackson Laboratories (Sacramento, California, USA). Mice arrived at the facility at approximately 4 weeks of age and were arbitrarily divided into 14 cages, each containing 3 mice (one individual of each strain per cage, following^24^).

Mice were housed in ventilated cages (32.5 cm × 17 cm × 14 cm; Ehret, Germany) containing aspen chip bedding (Jamieson’s Pet Food Distributors LTD), nesting material (cotton nestlet, Ancare, NY, USA; Enviropak nesting material, Datesand, UK), and a polycarbonate hut (Bio-Serv, NJ, USA). All animals were provided ad libitum food (irradiated Lab Diet Rodent Chow 2918) and reverse osmosis tap water. Mice were kept on a 12-h light cycle (lights on at 07:00 h) with controlled room temperature (mean ± SD; 20.7 ± 0.1 °C) and humidity (45.8 ± 4.7%). Cages were changed every two weeks by an experimenter familiar with the animals. Mice were handled with cupped hands or in their own inverted hut. Autoclaved pumpkin seeds and shredded coconut were sprinkled in the home cage for all mice at cage changing and for playpen mice upon returning from the playpen.

### Experimental design

**Figure 1:**
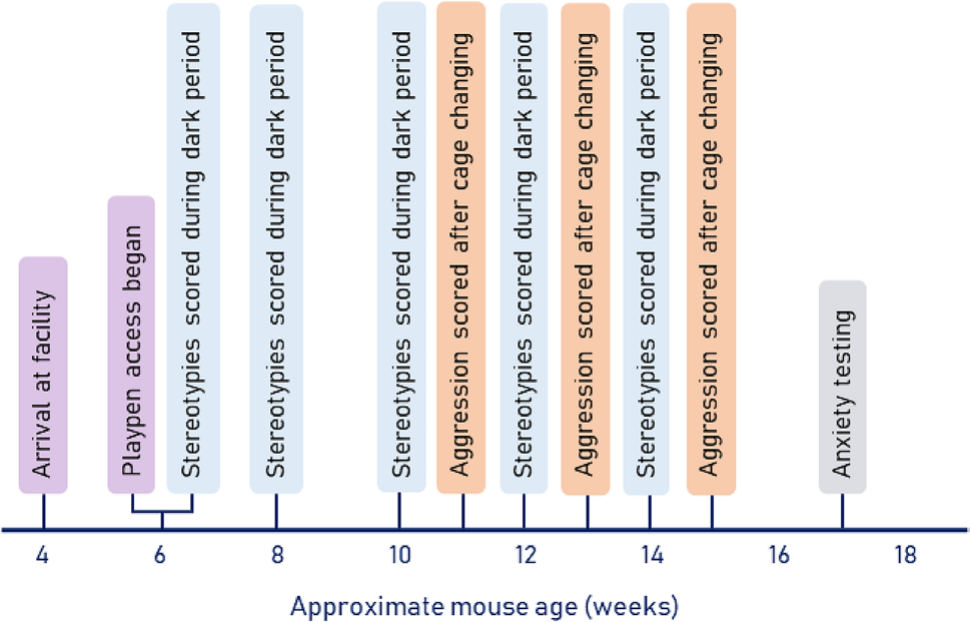
Experimental timeline for data collection and key events. Mice were given playpen access every week, beginning at six weeks of age. Stereotypies were scored during the dark period, and aggression was scored during the light period in the hour immediately following cage changing.

Mice (n = 42) housed in mixed-strain trios were assigned to treatments using a random number generator, with each number corresponding to playpen or control treatments for the cage (n = 7 mice per strain in each treatment; sample size is appropriately powered according to Mead’s rule^25^). Locations of individual cages on the cage rack were alternated according to treatment. Mice in the playpen treatment were given access 3 times per week for 30 min at a time, beginning at 6 weeks of age (± 3 days), and ending when mice were 17 weeks old (± 3 days). Control mice remained in their home cages. The timeline for data collection is outlined in Fig. 1.

### Playpens

Additional details on the playpens, including a photo, are provided in Ratuski et al.^23^. Briefly, the playpens consisted of two Optirat Plus cages (38.9 cm × 56.9 cm × 26.2 cm, Animal Care Systems, USA) connected by a PVC tunnel (5-cm diameter) inserted through the filter slot. One side contained soil (Garden Club 3-in-1 Mix, Canada) or coconut fibre (Thrive Natural Compressed Coconut Fiber Reptile Bedding, Petsmart, Canada) as burrowing substrate (substrate type was alternated between sessions), a triangular shelter made of black corrugated plastic, and a plastic upper mezzanine with an opaque elbow joint as a tunnel. Burrowing substrates were autoclaved before use and watered periodically to retain moisture. The other side of the playpen contained a running wheel (14 cm diameter; Kaytee Comfort Wheel, USA), a tunnel (Amber Mouse Tunnel, Bio-Serv, USA), climbing and shelter structures (Playmobil Park Playground and a cube structure made from magnetic PicassoTiles, USA), wood chip bedding, paper nesting material (Enviro-Dri, Shepherd Specialty Papers, USA), and plastic netting and hoops suspended from the lid for climbing.

To move mice in and out of the playpen, the home cage was placed next to the playpen, the cage lid removed, and an S-shaped tunnel (65 cm long, Tiny Tales Transport Tubes, Petsmart, Canada) was placed between the cage and the playpen. Mice had 5 min to cross the tunnel voluntarily; after 5-min, any remaining mice were transferred into or out of the playpen by the researcher using the inverted hut.

### Data collection

For home cage behaviour outcomes, the cages were video-recorded using infrared cameras (Amcrest, Texas, USA) connected to a DVR system (Amcrest ProHD 8CH Digital Recorder, Texas, USA). All videos were scored by observers blind to treatment and cage identity, with videos provided in randomized order for all outcomes. Observers could not be blinded to strain.

#### Stereotypies

Scoring methodology and ethogram for stereotypic behaviour (Table 1) were adapted from Novak et al.^26^ and Bailoo et al.^7^. Stereotypic behaviour was scored from videos using 0–1 scan sampling (15-s intervals for the first 15 min per h) during 6 h of the dark phase (between 20:00 and 02:00), resulting in 360 scans per observation day. We also recorded whether mice were active at any point during the 15-s scan; if mice were motionless for the entire scan they were scored as inactive. We used these scores to calculate stereotypies as a proportion of active time. Baseline was scored on one day when mice were 37–38 days old (before playpen access began), and subsequent scoring began one week later and continued every two weeks until mice were 14.5 weeks old (Fig. 1). Stereotypies were scored on both playpen and non-playpen days at each two-week interval, and these days were combined to create a mean value for each scoring period for a total of six periods scored per mouse (one baseline week and five periods after playpen access began).

**Table 1:** Ethogram for stereotypic and agonistic behaviour scoring.

Category	Behaviour	Description
Stereotypic	Bar-mouthing	Holding onto the bars of the wire lid with her forepaws and biting or mouthing along the bar for at least 3 s
	Circling	Following a circular path on the wire lid or cage floor (for at least 3 repetitions)
	Route-tracing	Following an invariant route on the wire lid or cage floor (for at least 3 repetitions)
	Twirling	Hanging from the cage lid with forepaws and spinning longitudinally in one location
	Backflipping	Pushing off the cage wall and flipping her body backwards
Agonistic	Pinning/boxing	Mouse pins down another mouse with forearms, or mice are on hind legs/sitting and pushing each other with forearms
	Chasing	Mouse closely pursues another mouse for > 1 s in duration
	Rough grooming	Mouse pins down another mouse with paws and vigorously grooms the other mouse; recipient appears stiff or trying to escape
	Mounting	Mouse attempting to mount and perform pelvic thrusts on another mouse
	Fighting	Mice are locked together, rolling around quickly, kicking, wrestling
	Displacement	Mouse pushes another mouse/supplants her from a resource (such as food, water, or nesting material)
	Anogenital investigation	Mouse persistently pushes or sniffs another mouse’s anogenital region

Inter-observer reliability for stereotypic behaviour was assessed using a Pearson correlation with 20% of the videos, each scored by two observers blind to treatment. Measures were excellent for both the total frequencies of stereotypic behaviour (r = 0.90), and number of observations where mice were scored as active (r = 0.97).

#### Aggression

Our ethogram to score aggression (Table 1) was adapted from Nip et al.^1^. Aggression was scored continuously from videos in the hour immediately following cage changing (an acute stressor) on three occasions spaced two weeks apart (at approximately 11.5, 13.5, and 15.5 weeks of age; Fig. 1). Inter-observer reliability for total rates of aggression was assessed from 20% of the videos (r = 0.87).

#### Anxiety

After three months of exposure to the playpens, anxiety behaviour was assessed using elevated plus maze and open field tests. The elevated plus maze was 25 cm high and consisted of two opposite open (25 cm × 5 cm) and two opposite closed arms (25 cm × 5 cm, with two walls 16 cm high) arranged in a cross shape with a square (5 cm × 5 cm) in the center. The open field apparatus was made of white acrylic glass (50 cm × 50 cm × 50 cm) with black lines that divided the floor area into 100 (5 cm × 5 cm) squares.

Mice were individually tested once in the elevated plus maze and then once in the open field on the following day. Within cages, mice were tested in a pre-assigned order, balancing strain testing order across cages and alternating between control and playpen cages. For both elevated plus maze and open field testing, the mouse was placed in the center of the apparatus and allowed to freely explore for 5 min. At the end of each trial, fecal boli were counted and the apparatus was cleaned with 70% ethanol. Behaviour was video-recorded (Canon VIXIA HF W10) and scored using Solomon Coder (Version 19.08.02) by an observer blind to treatment. Mouse location was determined based on the location of her head and shoulders.

Inter-observer reliability was assessed from 20% of the videos from each test, and was again excellent (elevated plus maze open arm time: r = 0.98, open field inner square time: r = 0.90).

#### Statistical analysis

Data analysis was conducted using SAS software (Version 9.4, SAS Institute Inc.) and plots were generated using the ggplot2 package in RStudio (R version 4.0.4) and Sigmaplot (version 15). Residual distributions were visually scrutinized. Covariance structures were selected based upon lowest Akaike’s Information Criterion values.

Stereotypies were analysed as proportion of active time. Six one-hour videos were missing or unusable, so there were six instances where a mouse’s average for an age were based upon five hours of observation instead of six. In preliminary analyses we identified no differences between playpen and non-playpen days, so type of day was excluded in subsequent analyses. Scored days were binned according to six ages spaced 1–2 weeks apart (see Stereotypies methodology). Mean frequencies of stereotypic behaviour were expressed as a percentage of active time during each scoring period. This measure was analysed using a repeated measures REML mixed model, testing the fixed effects of treatment, strain, age, and interactions of treatment*age, treatment*strain, strain*age, and treatment*strain*age with cage nested within treatment and mouse nested within treatment*strain as random intercepts.

For aggression in the home cage, results were analysed as mean frequencies in the hour following cage-changing on three days spaced two weeks apart. We used the same analysis as described above. For anxiety tests, we tested percent time spent in open arms of the elevated plus maze or inner squares of the open field as dependent variables, and analysed these using a mixed model with fixed effects of treatment, strain, a treatment*strain interaction, specifying strain as repeated within cages. One animal (a BALB/cJ mouse from the playpen treatment) was excluded from the elevated plus maze analysis because of an unusable video file.

## Results

### Stereotypies

**Figure 2:**
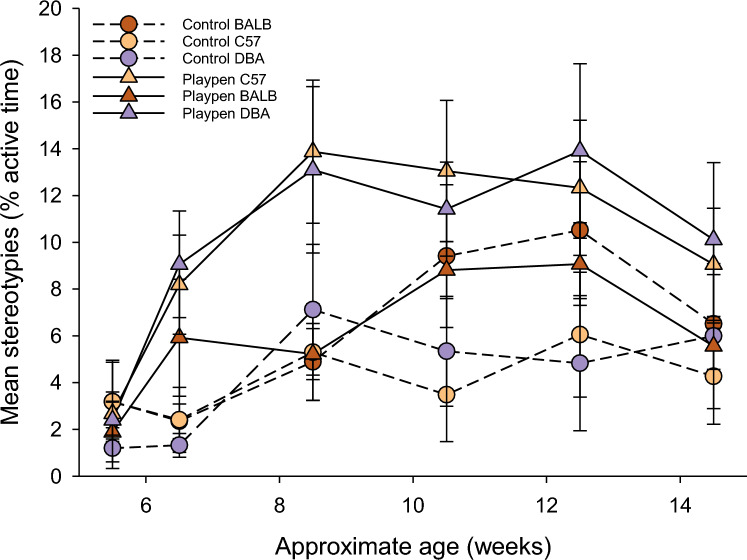
Mean % of active time spent performing stereotypies across six ages. The first timepoint represents baseline (i.e. before mice had experienced playpens). Data are shown as means and SE (n = 21 mice per treatment, with 7 mice per strain within treatment). Note that analysis was performed on transformed values but untransformed values are shown here.

There was a significant interaction between treatment and age (F_5,180_ = 3.73, *p* < 0.01). Mice from the playpen and control groups displayed similar levels of stereotypic behaviour at baseline, and diverged after 8 weeks of age, such that mice with access to the playpen spent more of their active time engaged in stereotypic behaviours in comparison to control mice who did not have access to the playpens (Fig. 2; F_1,12_ = 11.02, *p* < 0.01); this difference was more notable for C57BL/6J and DBA/2J mice, but the interaction between strain and treatment was not significant (F_2,24_ = 2.33, *p* = 0.12). During baseline observations, mice had similar rates of stereotypic behaviour (3.2% ± 0.98 for control mice and 3.1% ± 0.77 for playpen mice; Fig. 2). Stereotypic behaviour started to diverge between treatments after the playpen treatment began, with playpen mice displaying more stereotypies at a younger age.

The form of stereotypic behaviour tended to vary by strain. Bar biting was often performed by BALB/cJ and DBA/2J mice, circling on the wire lid was generally performed by C57BL/6J mice, and twirling was generally performed by DBA/2J mice. Route tracing and backflipping were rarely observed (Table 2). Mice in the two treatments showed similar levels of activity; of the 60 scans/observational day, control mice were active for an average of 47.9 ± 0.54 scans, while playpen mice were active for 45.9 ± 0.60.

**Table 2:** Prevalence of individual stereotypic and aggressive behaviours. Data are shown as raw means ± SE according to treatment and strain (n = 7 mice of each strain per treatment), and are presented for descriptive purposes only. Data are from age 10 weeks and up. Stereotypies are shown as a % of active time; aggression values represent average frequencies from three 1-h observations after cage changing. Strain designations are abbreviated within the table

Category	Behaviour	Strain	Playpen	Control
*Stereotypies (% of active time)*	Barbiting	BALB	9.3 ± 1.00	8.6 ± 1.40
		C57	0.6 ± 0.16	0.3 ± 0.13
		DBA	5.1 ± 0.93	2.5 ± 0.50
	Circling	BALB	0.2 ± 0.09	0.5 ± 0.26
		C57	13.7 ± 1.50	5.2 ± 1.23
		DBA	0.1 ± 0.03	0.3 ± 0.11
	Twirling	BALB	0.0 ± 0.02	0.0 ± 0.00
		C57	0.2 ± 0.11	0.0 ± 0.01
		DBA	9.9 ± 2.03	3.8 ± 1.54
	Route tracing	BALB	0.5 ± 0.34	0.1 ± 0.03
		C57	0.2 ± 0.08	0.1 ± 0.07
		DBA	0.0 ± 0.01	0.2 ± 0.13
	Backflipping	BALB	0.1 ± 0.09	0.0 ± 0.00
		C57	0.0 ± 0.00	0.0 ± 0.00
		DBA	0.0 ± 0.02	0.0 ± 0.00
*Aggression (mean frequency)*	Anogenital sniffing	BALB	3.0 ± 0.50	2.4 ± 0.35
		C57	6.4 ± 0.95	9.6 ± 1.20
		DBA	3.6 ± 0.56	3.5 ± 0.66
	Chasing	BALB	1.0 ± 0.26	0.4 ± 0.18
		C57	1.3 ± 0.25	2.7 ± 0.84
		DBA	0.1 ± 0.07	0.5 ± 0.20
	Displacement	BALB	0.8 ± 0.24	0.2 ± 0.09
		C57	1.2 ± 0.27	1.1 ± 0.30
		DBA	0.7 ± 0.21	0.7 ± 0.21
	Pinning/boxing	BALB	0.2 ± 0.10	0.2 ± 0.09
		C57	0.8 ± 0.20	1.7 ± 0.48
		DBA	0.2 ± 0.09	0.5 ± 0.21
	Rough grooming	BALB	0.0 ± 0.00	0.0 ± 0.00
		C57	0.1 ± 0.08	0.7 ± 0.30
		DBA	0.1 ± 0.10	0.1 ± 0.07
	Mounting	BALB	0.0 ± 0.00	0.1 ± 0.05
		C57	0.1 ± 0.01	0.5 ± 0.26
		DBA	0.0 ± 0.00	0.0 ± 0.00
	Fighting	BALB	0.1 ± 0.05	0.1 ± 0.05
		C57	0.1 ± 0.05	0.1 ± 0.05
		DBA	0.0 ± 0.00	0.0 ± 0.00

### Aggression

**Figure 3:**
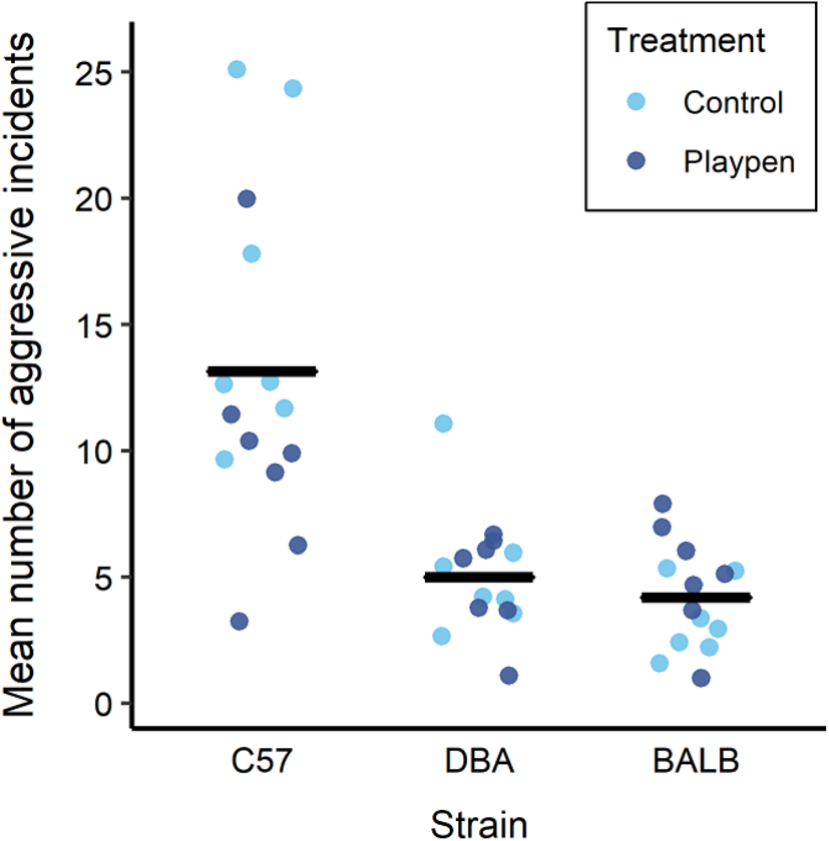
Average number of aggressive incidents observed after cage changing, shown separately by strain. Points represent the average per mouse from 3 observation days. Black bars represent strain means. Each treatment consisted of 21 mice, with 7 mice per strain.

Anogenital sniffing was the most common agonistic behaviour; more overt forms of aggression such as fighting were rarely observed (Table 2). There was an effect of strain (F_2,24_ = 22.85, *p* < 0.0001), driven by higher rates of aggression among the C57BL/6J mice (Fig. 3). We also found an interaction between treatment and strain (F_2,24_ = 3.86, *p* = 0.03), driven by a reduction in aggression for C57BL/6J mice in the playpen treatment, but no such reduction for the other strains. There was no effect of age, or interaction with age and treatment or strain.

### Anxiety

**Figure 4:**
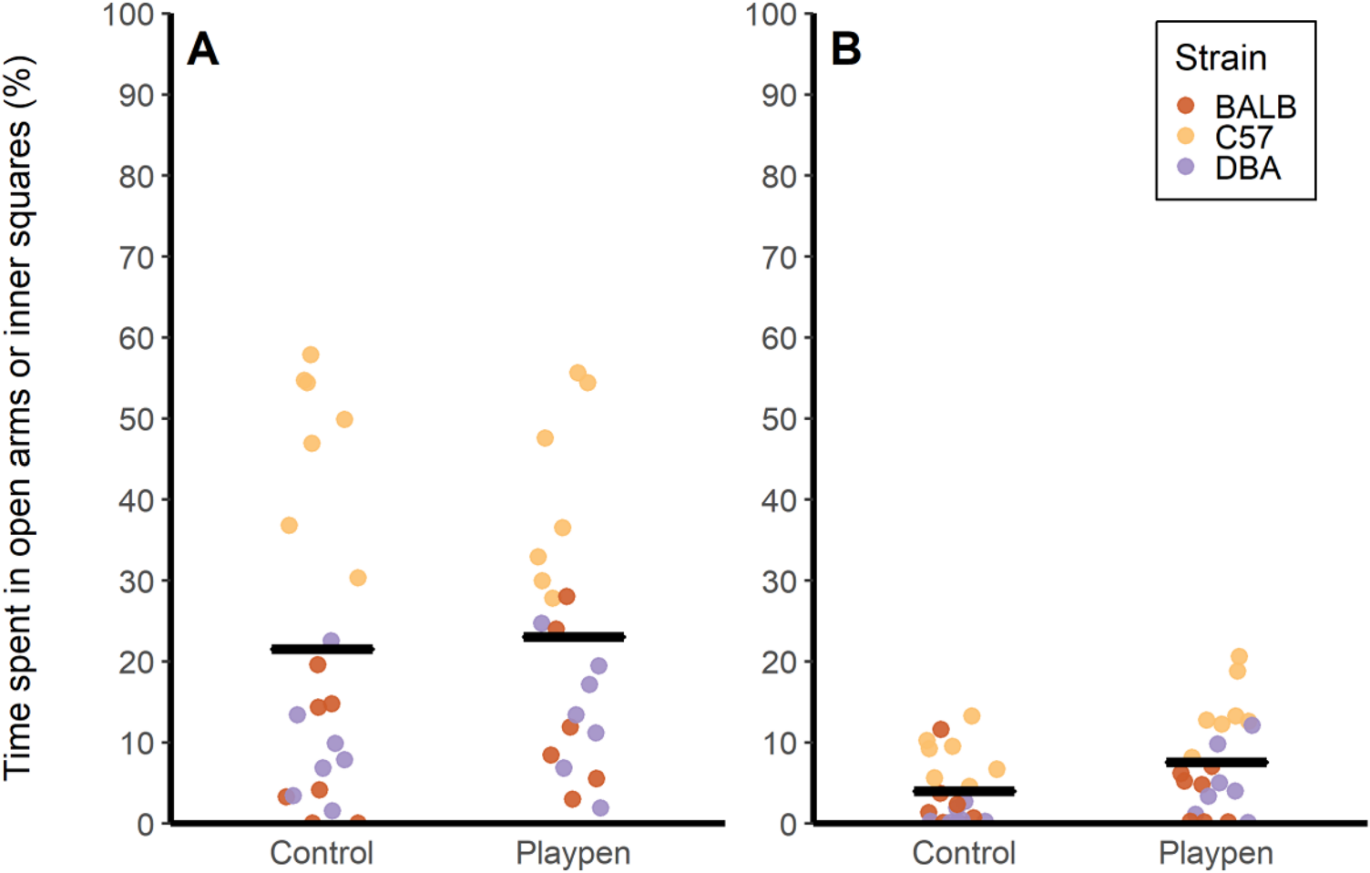
Time (%) spent in open arms of elevated plus maze (panel **A**) and inner squares of open field (panel **B**). Points represent individual mice. Trials were 5 min long. n = 21 mice per treatment.

Treatment did not affect time spent in open arms of the elevated plus maze (F_1,12_ = 0.12, *p* = 0.73), but this measure was affected by strain (F_2,23_ = 58.17, *p* < 0.0001), with C57BL/6J mice spending more time in the open arms of the elevated plus maze compared to the other two strains (Fig. 4). Compared to controls, mice in the playpen treatment spent slightly more time in the inner squares of the open field (7.5% ± 1.3 vs. 4.0% ± 0.9; F_1,12_ = 10.41, *p* = 0.01). There was also an effect of strain (F_2,24_ = 25.56, *p* < 0.0001) on time spent in the inner squares (Fig. 4), with C57BL/6J mice spending more time in the center of the open field. Interactions between strain and treatment were not significant.

Mice provided access to the playpens produced fewer fecal boli than control mice in the elevated plus maze (2.6 ± 0.7 vs. 4.7 ± 0.9, respectively; F_1,12_ = 12.15, *p* = 0.005), but not in the open field (F_1,12_ = 1.89, *p* = 0.19). There were also strain differences in fecal boli output in elevated plus maze testing (F_2,23_ = 53.31, *p* = 0.0001). C57BL/6J mice did not produce any fecal boli in the elevated plus maze regardless of treatment; BALB/cJ mice produced the most (7.8 ± 0.9), followed by DBA/2J mice (3.4 ± 0.6). In the open field, only strain had a significant impact on fecal boli output (F_2,24_ = 14.18, *p* < 0.0001). BALB/cJ mice produced the most fecal boli (7.8 ± 1.4), followed by DBA/2J (2.1 ± 0.8) and C57BL/6J mice (0.8 ± 0.6).

## Discussion

Contrary to our predictions, mice with playpen access displayed more stereotypic behaviour than did control mice. We propose three interpretations for the increased stereotypic behaviour in playpen mice, based on possible underlying mechanisms: (1) stereotypies are indicative of negative emotional states, and these states were more prevalent in the playpen mice; (2) stereotypies reflect a more active coping attempt by mice^27^, and the playpen promoted this more active coping response; (3) stereotypies indicate heightened motivation to escape the home cage, and mice were more motivated to escape after their playpen experience. Regardless of the interpretation, these results suggest that playpen access (as provided in the current study) was not enough to prevent the development of stereotypies.

Animals experiencing a negative contrast (i.e., receiving a downshift in reward after being exposed to more valuable resources) may experience negative emotions (e.g.^28^). In particular, a loss of environmental complexity can negatively impact rodent welfare. For example, one study demonstrated that when mice were transferred from more complex cages into barren cages, they exhibited more negative emotional states, neurological signs of stress, and increased vulnerability to drug addiction^29^. Latham and Mason^30^ observed increased stereotypic behaviours in mice that lost access to environmental enrichment, and proposed that these behavioural changes were due to feelings of frustration resulting from the downshift in environmental quality. However, another study did not see increased stereotypies following removal of enrichment from adult mice, suggesting that early life environmental enrichment had neuroprotective effects^6^. In hamsters, removal of enrichment items resulted in more pessimistic responses in a judgment bias task^31^. Enrichment removal also results in higher sensitivity to reward loss in rats^28^, and has been used to model significant loss, resulting in behavioural and neurological indicators of depression^32,33^. Therefore, it is possible that returning to shoebox cages after temporary playpen access was perceived as a negative contrast by the mice, thereby inducing negative emotional states that were expressed through increased stereotypic behaviour. This effect may be particularly concerning for welfare if playpen access is infrequent or permanently discontinued.

Alternatively, it is possible that playpen access provided mice more agency, allowing them to better develop coping mechanisms (in line with the ‘coping hypothesis’ of stereotypic behaviour^34^). The home cage may have been perceived equally by mice in both treatments, but playpen mice better developed active coping mechanisms (in the form of stereotypies) to deal with the stress of the shoebox cage. It has been suggested that stereotypies initially develop as a coping strategy to reduce negative impacts of stressful stimuli^27^. Although stereotypies may begin as an active coping mechanism, there is no clear link between the performance of all stereotypies and the reduction of stress^35^, and limited evidence in support of the benefits of stereotypical coping once behaviours have become established^36^. However, there is a negative correlation between stereotypic behaviour and time spent inactive-but-awake, suggesting that stereotypic behaviour is a form of hyperactivity in response to stress, rather than the alternative of hypoactivity or depression-like behaviour^3,35^. We attempted to score inactive-but-awake behaviour from videos but were unable to reliably capture this behaviour. Barbering behaviour could also be indicative of stressful living conditions, but we did not observe any barbering in our mice.

As a third interpretation of stereotypic behaviour, mice may have been highly motivated to return to the playpens (or increasingly motivated to remove themselves from the shoebox cage environment) and perhaps developed stereotypies as a result. Our previous work indicates that mice perceived playpens positively, with decreasing latencies to enter and increased anticipatory behaviour prior to access^23^. After 9 days of playpen access, almost all mice were running to voluntarily enter playpens, demonstrating their motivation to gain access. These findings would support stereotypy development as an escape-motivated behaviour. In addition to increased stereotypic behaviour, Latham and Mason^30^ also reported increased motivation to gain access to enrichment resources in mice that were downgraded from complex to standard housing. Bar biting in particular is thought to reflect the motivation to escape the cage^37,38^; this was a commonly exhibited behaviour in our mice.

Our results support previous work showing that stereotypies increase as mice age into adulthood. Würbel et al.^39^ found that stereotypies occupied 7.4–10.7% of active time when mice reached 100 days of age; in the current study, C57BL/6J and DBA/2J mice with playpen access surpassed this rate (Fig. 3). While stereotypic behaviour itself is an unreliable welfare indicator, environmental circumstances resulting in stereotypic behaviour are typically linked with other indices of poor welfare^40^. Rates of stereotypic behaviour have variable relationships with emotional states^26,41^ and hormonal indicators of stress^42^. Non-stereotyping individuals living in deficient environments may actually be experiencing greater suffering than those expressing high rates of stereotypic behaviour, highlighting a need for consideration of other welfare indicators in assessments^40^. Multiple factors such as the strain of the mouse and the form of stereotypy displayed may be important when considering the relationship between stereotypic behaviour and welfare^26,41^.

Cage changing can trigger aggressive interactions^15^, and potentially increase territorial behaviour if mice perceive the change in environmental conditions as a competitive scramble for resources^12^. Overt or injurious aggression is uncommon in female laboratory mice; agonistic behaviour is more often expressed through behaviours such as anogenital investigation, displacement, and chasing^1^. Anogenital investigation was the most common form of agonism seen in our mice. As we also saw when mice were inside the playpens^23^, the C57BL/6J mice were most likely to act aggressively in the home cage. There was also an effect of treatment on this strain in the current study; C57BL/6J mice with playpen access performed less aggressive behaviour in the home cage than control C57BL/6J mice, indicating that these mice were more resilient to the stress of cage changing. The other two strains were generally the recipients of aggression, rather than the instigators, regardless of treatment. C57BL/6J mice have been classified as a highly social strain of mice^43,44^, but this may be due to a heightened motivation to exert dominance over other mice. This result suggests housing other strains with C57BL/6J mice may be a welfare concern, as social behaviour may be altered by mixed-strain housing^45^, and subordinate mice may suffer from chronic stress resulting from persistent aggression^46^. Importantly, it also adds to the body of evidence studying enrichment-related agonism between female laboratory mice.

Our results indicate that regular playpen access decreased some measures of anxiety in mice, with notable differences between strains. Playpen mice defecated less than control mice in the elevated plus maze. C57BL/6J mice also averaged four times more time in the open arms of the elevated plus maze and three times more time in the center of the OF compared to the other strains tested, with no fecal boli produced in the elevated plus maze test. Previous studies have also found differences between strains in anxiety behaviour phenotyping^47–49^. In line with our findings, one previous study has suggested that C57BL/6J mice cope with the anxiogenic conditions of these tests in a different way than BALB/cJ mice, exhibiting more locomotory behaviour, while BALB/cJ mice typically repress locomotion in these environments and tend to defecate more^49^.

Reduced defecation by playpen mice is consistent with reduced anxiety^50^, but the effect of treatment was not consistent across the two test procedures (playpen access positively influenced open field but not elevated plus maze behaviour) or measures (defecation was influenced in the elevated plus maze only). Both tests are commonly used as indicators of rodent anxiety, but differ in sensitivity and reliability; time spent in the open arms of the elevated plus maze may be a more reliable indicator of anxiety behaviour than time spent in the inner squares of the open field^51^. It has also been suggested that the elevated plus maze is more anxiogenic than the open field^49^. The inconsistency between tests in the current study may indicate that playpen access affects other aspects of behaviour measured in the open field, such as locomotory activity or exploration^50^; i.e., playpen access may make mice more motivated to explore rather than actually reducing anxiety. We did not measure hormonal outcomes related to stress in the present study, but this may have helped to interpret our behavioural findings. Exposure to a larger and more complex environment three times per week has been demonstrated to reduce the impacts of stress on hormonal and immune system activation in rats^52^.

In this study, we only tested female mice and therefore cannot draw conclusions about how male mice might have been influenced by temporary playpen access; in particular, male mice are known to have different forms and rates of aggressive behaviour in response to environmental changes compared to female mice^12^. Additionally, our interpretation of stereotypic behaviour and its implications for animal welfare are inconclusive. Future studies should consider measures of affective states within the home cage to help interpret differences in stereotypic behaviour. Lastly, mice in our study had access to one particular design of playpen, and access was limited to three times per week for 30 min sessions during the light phase. Effects may differ with other playpen designs and access routines.

## Conclusions

Playpens provide laboratory mice with temporary access to more space and environmental complexity, increasing opportunities for natural behaviour. However, contrary to our predictions, playpens did not mitigate the development of stereotypic behaviour expressed in the home cage, with playpen mice demonstrating more stereotypies than controls. Playpen access resulted in strain-dependent improvements in some measures of aggression and anxiety. There were notable differences between strains in several outcomes, highlighting the importance of considering multiple genetic strains of mice in behavioural work. Further work is required to better understand how different access routines and forms of temporary environmental complexity affect the welfare of mice living in shoebox cages.

## Data Availability

All data generated or analysed during this study are available via Figshare. 10.6084/m9.figshare.25114157
