# Author Correction: Effects of temporary access to environmental enrichment on measures of laboratory mouse welfare

**DOI:** 10.1038/s41598-024-69327-1

**Published:** 2024-08-12

**Authors:** A. S. Ratuski, L. Améndola, I. J. Makowska, D. M. Weary

**Affiliations:** UBC Animal Welfare Program, Faculty of Land and Food Systems, University of British Columbia, Vancouver, Canada

Correction to: *Scientific Reports* 10.1038/s41598-024-65480-9, published online 02 July 2024

The Ethics declarations section in the original version of this Article contained an error.

“Animal Welfare Institute had a role in design, analysis, or interpretation of LA’s portion of the study.”

now reads:

“Animal Welfare Institute had no role in design, analysis, or interpretation of LA’s portion of the study.”

In addition, Reference 18 was incorrectly given as:

Makowska, I. J., Franks, B., El-Hinn, C., Jorgensen, T. & Weary, D. M. Standard laboratory housing for mice restricts their ability to segregate space into clean and dirty areas. *Sci. Rep.* **9**, 6179. 10.1038/s41598-019-42512-3 (2019).

The correct reference is listed below:

Makowska, I.J. & Weary, D.M. A good life for laboratory rodents? *ILAR J* **60**, 373–399. 10.1093/ilar/ilaa001 (2019).

The original Article has been corrected.

